# Case Report: Intraoperative detection of a rare superior vena cava variant in chest wall intravenous port implantation

**DOI:** 10.3389/fsurg.2025.1610944

**Published:** 2025-08-29

**Authors:** Shaofeng Yang, Jing Du, Donghai Li

**Affiliations:** Department of Thyroid Breast Surgery, Inner Mongolia Medical University, Affiliated Hospital of Inner Mongolia Medical University, Inner Mongolia, Hohhot, China

**Keywords:** infusion port, venous variant, persistent left superior vena cava (PLSVC), chest wall port

## Abstract

As a fully implantable central venous infusion device, venous access port (VAP) is widely used in long-term tumor chemotherapy and parenteral nutrition due to its long maintenance cycle and high patient comfort, and is usually divided into upper arm port and chest wall port. Chest wall port is usually implanted by internal jugular vein or subclavian vein access, and the device is placed in a pocket in the chest wall subcutaneously, which is a widely used totally implantable venous access port (TIVAP) in recent years, and has the advantages of long retention time and low complication rate compared with peripherally inserted central venous catheter (PICC). Here, we report a case of incidental diagnosis of persistent left superior vena cava (PLSVC) during the implantation of chest wall IV port after rectal cancer surgery. In patients undergoing IV port implantation, congenital venous variations were found to commonly result in ectopic catheter ends. Postoperative catheter malfunction, thrombosis, and cardiac arrhythmias have all been associated with catheter tip location, and accordingly, we conducted a comprehensive review of this case with the aim of improving the safety of infusion port implantation inpatients.

## Introduction

With the aging of the population, cancer has become the leading cause of death (1). However, the treatment of cancer is very complex, and chemotherapy has become one of the main ways to treat cancer in addition to surgery (2). However, most chemotherapy drugs are cytotoxic and peripheral intravenous injection can cause vascular damage, so it is important to establish good venous access (3). As a new type of infusion, TIVAP (totally implantable venous access port) moves from the internal jugular vein to avoid the damage to the blood vessels caused by chemotherapy drugs. However, this infusion method has high requirements for the patient's vascular condition, and the diameter of the internal jugular vein wall and the direction of the blood vessel determine whether the infusion device can be successfully implanted. This case reports a patient with vascular malformation, which has a guiding role in the placement of infusion ports in the future, and enriches the gap in the field of infusion port implantation in patients with developmental abnormalities.

**Figure 1:**
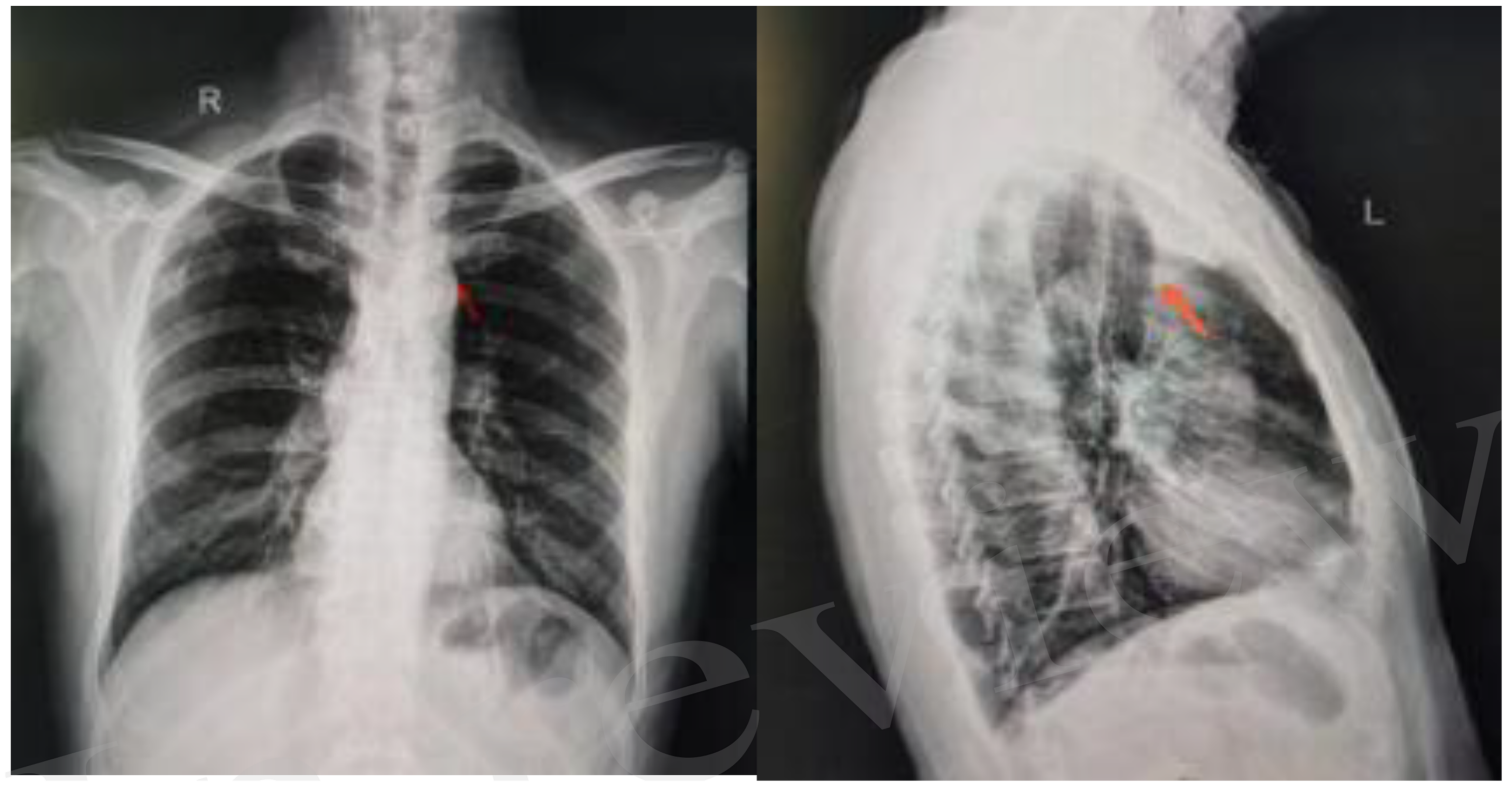
Chest x-ray in the frontal position of the chest with the catheter end under the 1st anterior rib on the left side lateral chest view (right) catheter ends at the lower edge of the 1st anterior rib on the left side.

**Figure 2:**
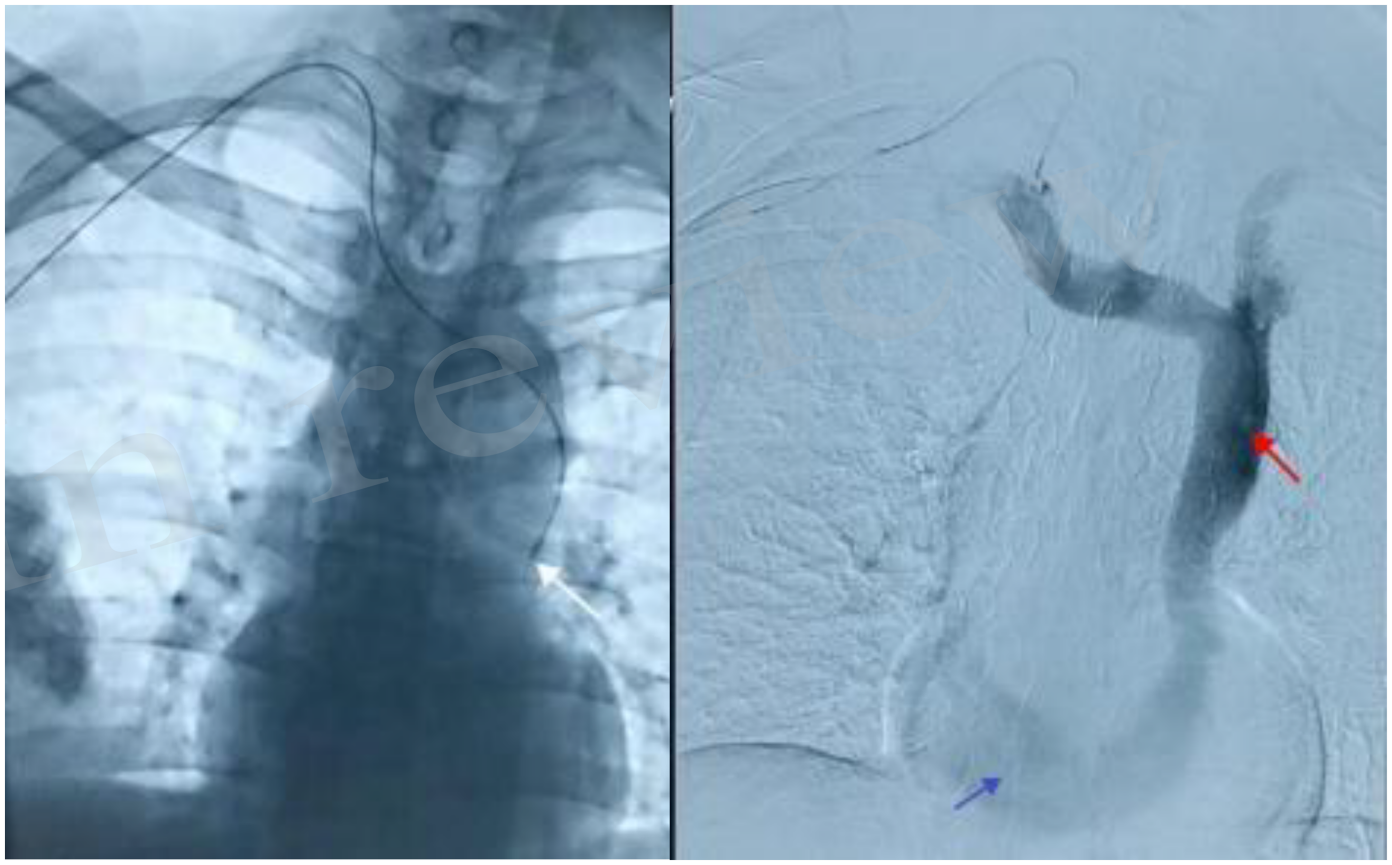
Final position of the catheter at the lower edge of the 6th anterior rib angiogram showing left superior vena cava (red arrow) and widened coronary sinus (blue arrow).

**Table 1:** Primary diagnosis of the patient and follow-up time.

0 day after colonoscopy was performed	7 days after colonoscopy was performed	10 days after colonoscopy was performed	29 days after colonoscopy was performed	51 days after colonoscopy was performed	74 days after colonoscopy was performed
Electronic colonoscopy—rectal cancer?	Laparoscopic anterior rectal resection Mesenteric lymph node dissection	Postoperative freezing: differentiated adenocarcinoma in the rectum invades the adventitia, and 1/12 of the lymph nodes are metastasized	Place an intravenous port and administer the first adjuvant chemotherapy.	A second adjuvant chemotherapy is given	Third adjuvant chemotherapy was given
77 days after colonoscopy was performed	82 days after colonoscopy was performed	120s day after colonoscopy was performed	141 days after colonoscopy was performed	168 days after colonoscopy was performed	189 days after colonoscopy was performed
Because chemotherapy has a decrease in the three lines, infection and fever, symptomatic treatment is given	After the fourth adjuvant chemotherapy, the patient had a post-chemotherapy reaction, and the white blood cells were reduced to 2*109	The fifth adjuvant chemotherapy was performed.	Sixth adjuvant chemotherapy was performed	Seventh adjuvant chemotherapy was performed	Eighth adjuvant chemotherapy was performed. After chemotherapy, a whole body examination was performed to evaluate the effect of chemotherapy, and the results of abdominal CT, magnetic resonance imaging, and blood tests showed that the patient's tumor disappeared and there was no metastasis to the peritoneum and other organs.
2025-3-21	2025-6-29				
At the first follow-up after surgery, the patient said that the shape, color, and frequency of defecation were normal, no weight loss, and he had recently gained 3 kg.	At the second follow-up after surgery, the patient said that his weight had increased by 7 kg from the postoperative period, there was no change in the shape of his bowel movement, the physical examination was normal, and there was no abnormality in the examination				

## Case report

The patient was a 57-year-old male, who presented with decreased stool frequency with blood in stool in January 2024, and was diagnosed with rectal adenocarcinoma by electronic colonoscopy in April 2024, and underwent laparoscopic anterior proctocolectomy + mesenteric lymph node dissection, and the postoperative pathology was staged as moderately differentiated adenocarcinoma, stage ⅢB, pTNM = pT3N1aMx0, based on which the XELOX chemotherapy regimen was formulated. pT3N1aMx0, according to the patient's tumor stage, the XELOX chemotherapy plan was formulated, and in order to perform postoperative adjuvant therapy, an IV port was implanted as a long-term drug delivery device. The patient's laboratory tests were normal and there were no absolute contraindications to surgery, so he underwent IV port implantation on May 7, 2024, with aright-sided approach, ultrasound localization of the right internal jugular vein, and ultrasound out-of-plane guided puncture, Place the deep vein catheter approximately 13 cm. After implantation of the chest wall IV port, a chest x-ray was performed to locate the end of the port catheter, which indicated that the end of the port was ectopic to the left lower edge of the first anterior rib, and could not be used normally. It could not be used normally.

And the patient complained of postoperative pain in the operative area and the right upper arm, accompanied by a feeling of suffocation in the precordial area. Under DSA guidance, the catheter was retracted, and after injection of contrast, the patient's right superior vena cava was found to be absent, and the contrast diffused along the left superior vena cava into the wide coronary sinus, and partly retrogradely into the right internal jugular vein (brachiocephalic vein), but not into the right atrium, so it could be judged that this patient was a rare venous variant of a persistent left superior vena cava (PLSVC) with an absence of right superior vena cava, and the final adjustment of the depth of the tip of the catheter was located in the left 6th anterior rib inferior border. The patient underwent 3 cycles of postoperative oxaliplatin sedation combined with capecitabine oral chemotherapy, and there was blood return during the use of the intravenous infusion port, with no catheter-related complications, and no other significant discomfort was reported (Table 1).

## Discussion

Persistent left superior vena cava (PLSVC) is a rare body vein anomaly with an incidence of about 0.5% in the general population. Due to the gradual degeneration and thinning of the proximal left anterior main vein during the embryonic period, the left atrial oblique vein is formed. If the degeneration process is incomplete, the left atrial oblique vein is abnormally widened, resulting in a remnant of left superior vena cava, which ultimately drains into the right atrium as a compensatory collateral branch through the widened coronary sinus (4, 5). The right internal jugular vein has a simple alignment with the superior vena cava, with a thick diameter, and the catheter is not easy to bend and angle. According to the relevant literature, it is recommended that the right internal jugular vein be chosen as the puncture vessel for thoracic wall infusion port implantation, and the ideal position of the catheter tip ranges from the middle and lower 1/3 of the superior vena cava to the superior vena cava-right atrium junction (CAJ) (6).

In this patient, the right superior vena cava was absent, and the right and left cephalic veins converged to the left superior vena cava, and the abnormal venous blood eventually flowed into the right atrium, with no abnormal changes in hemodynamics, and there were no obvious clinical symptoms and signs, which had an impact on the patients who engaged in strong physical labor and required large amounts of rapid rehydration, while the rest of the patients would not be detected and did not need treatment. However, due to its abnormal venous route, it may cause difficulties in routine implantation of catheters in the infusion port, as well as poor control of the length of the guidewire during the operation, which may induce arrhythmia by mechanical stimulation of the heart. Therefore, preoperative examination should be performed routinely to clarify the presence of this abnormality. Preoperative assessment of the positioning of the catheter so that the operator in the implantation of the catheter before the choice of the appropriate access and catheter length selection, to prevent catheter end ectopic may lead to postoperative catheter dysfunction, the catheter end of the catheter end is too deep will lead to arrhythmia, catheter-related right atrial thrombosis, the catheter end of the catheter end position is too shallow easily adhered to the vascular wall or the occurrence of ectopic catheter to the internal jugular vein, the use of the process of pumping back to the absence of blood return, unable to administer drugs.

## In conclusion

This case underscores the critical role of preoperative radiologic evaluation in venous access planning, particularly for patients with congenital vascular anomalies. While the incidence of persistent left superior vena cava (PLSVC) is rare (0.3%–0.5% in the general population, rising to 3%–10% in congenital heart disease cohorts), its unanticipated discovery during Totally Implantable Venous Access Port (TIVAP) placement poses significant technical challenges. Our experience aligns with literature reports that PLSVC-associated anomalies—including guidewire misdirection, catheter coiling, or aberrant tip positioning—can lead to procedural failure or complications if not anticipated. Comparative Context with Published PLSVC Cases: Our findings resonate with key themes in contemporary PLSVC literature: (1) Access Route Dilemma: Similar to cases reported in Cureus (7) and The Journal of Vascular Access (8), we encountered profound difficulty advancing the guidewire centrally via the right cephalic vein. PLSVC typically drains into the coronary sinus, creating an acute angle that impedes guidewire/catheter passage into the right atrium—a common challenge highlighted across case series. (2) Imaging Imperative: As emphasized in recent reviews, preoperative detection is paramount. While routine echocardiography for all TIVAP candidates remains impractical, our case reinforces recommendations for targeted imaging in patients with: Failed initial access attempts. Unexplained procedural resistance/pain. Known congenital syndromes or cardiac anomalies. Contrast-enhanced CT or MR venography, as utilized here and supported by cases in CardioVascular and Interventional Radiology (9), offers the most reliable roadmap for anomalous venous anatomy, enabling proactive surgical planning (It should be noted here that the CT, MRI, and echocardiography we refer to the tests that need to be done in patients with the above conditions, not the routine tests for all patients with IV implants.). Safety and Complication Avoidance: Our caution against aggressive manipulation of wires reflects the urgent advisories found in the PLSVC literature concerning the risk of perforation of the SVC (10). The utilization of interventional radiology, whether as a backup or primary method, as indicated in this context, is becoming increasingly recommended in cases of complex anatomy to improve safety. Despite the unanticipated nature of the patient's anatomy, the author team concluded that the right-sided approach was appropriately employed, and that the introduction of a new left-sided approach in light of the patient's trauma would likely exacerbate both the trauma and pain experienced. Moreover, during the interventional guidance and imaging examinations, we observed that the patient's superior vena cava is positioned above the left side of the heart, and that the bilateral internal jugular veins are present and in good condition, continuing bilaterally into the left superior vena cava which drains into the heart.

At the same time, patients demand that under the premise of pursuing tumor treatment, the principle of reducing trauma and pain is the principle.

This case suggests the importance of radiologic techniques in the selection of surgical access to the IV port and the detection of congenital anomalies. Surgeons placing IV ports should have an in-depth understanding of the possible complications and potential anatomical variations of the implantation process, perform preoperative examinations to clarify the vascular conditions of the surgical site, choose the optimal placement route for the patient to minimize the patient's pain and risk, and anticipate the causes of implantation difficulties during the operation, and place the ports safely with the help of imaging technology under direct vision if necessary (Figures 1, 2). Patients who need to apply fully implantable intravenous drug delivery are usually patients with tumors that require long-term chemotherapy. Echocardiography can be used for preoperative assessment of implantation in patients undergoing neoadjuvant chemotherapy, and computed tomography with enhancement scanning and magnetic resonance technology can also provide a more reliable diagnosis of abnormalities of the superior vena cava, and for postoperative adjuvant chemotherapy, the location of the access vessels can be determined based on the patient's first preoperative examination. To determine the location of the access vessels and the surrounding adjacent structures, the location of the access vessels and the surrounding adjacent structures can be determined according to the patient's first preoperative examination. Although the variation of the vein causes great difficulties in the placement of the infusion port, considering that the probability of occurrence of this case is quite small, it is not recommended to cover all patients with different individuals, so it is not recommended to perform preoperative 2D echocardiography in patients who need TIVAP.

When guidewire access disorder occurs, consideration should be given to the superior vena cava for compression, vascular stenosis, and deformity of vascular orientation may lead to rupture of the superior vena cava and risk of bleeding. When guidewire access disorder occurs, consideration should be given to the superior vena cava for compression, vascular stenosis, and deformity of vascular orientation. Any resistance should remind the surgeon that the patient should not be implanted with conventional surgical procedures, and that interventional surgery (Interventional therapy is a minimally invasive treatment that utilizes modern high-tech methods. This involves the use of specialized catheters, guide wires, and other precise instruments, which are introduced into the human body under the guidance of medical imaging equipment, for the diagnosis and localized treatment of pathological conditions within the body.) should be performed if necessary to make the surgery safer. For the late-stage patients, we followed up, and by 2025-06-22, the patients had completed the relevant cycles of chemotherapy, and the infusion port was regularly maintained, and dark red blood was seen in the retraction, and the use was unobstructed.

The placement of the port is relatively safe, which reduces the risk of catheter misplacement into other blood vessels and greatly reduces the probability of bleeding. However, for unmarried and childless patients, interventional surgery may lead to resistance from some patients. But most patients say that they prioritize addressing the distress caused by the tumor rather than worrying about the effects of radiation dose on fertility.

At present, there are few reports of persistent left superior vena cava (PLSVC) variations in China and abroad, and there is no consensus on the choice of access and depth of the catheter tip for the implantation of a port in a persistent left superior vena cava (PLSVC). This case report has some implications for patients who encounter abnormal sheath and guidewire entry during port implantation and for patients who experience abnormal postoperative pain, as preoperative evaluation can prevent unplanned port removal and ensure safe catheter use.

Last, For patients with anatomical variations, any resistance to the surgical process during surgery should indicate the intention of surgery to consider whether the patient has anatomical variations, and further reasonable examinations should be performed to enhance surgical safety.

## Data Availability

The original contributions presented in the study are included in the article/Supplementary Material, further inquiries can be directed to the corresponding author.
